# Different gender-derived gut microbiota influence stroke outcomes by mitigating inflammation

**DOI:** 10.1186/s12974-022-02606-8

**Published:** 2022-10-04

**Authors:** Jinchen Wang, Yi Zhong, Hua Zhu, Omer Kamal Mahgoub, Zhihong Jian, Lijuan Gu, Xiaoxing Xiong

**Affiliations:** 1 Department of Neurosurgery, Renmin Hospital of Wuhan University, 99 Zhang Zhidong Rd, Wuhan, 430060 Hubei China; 2 Department of Anesthesiology, Zhujiang Hospital of Southern Medical University, Guangzhou, China; 3 Central Laboratory, Renmin Hospital of Wuhan University, Wuhan, China

**Keywords:** Sex differences, Fecal microbiota transplantation, Gut microbiota, Ischemic stroke, Inflammation

## Abstract

**Background and purpose:**

Stroke is associated with high disability and mortality rates and increases the incidence of organ-related complications. Research has revealed that the outcomes and prognosis of stroke are regulated by the state of the intestinal microbiota. However, the possibility that the manipulation of the intestinal microbiota can alter sex-related stroke outcomes remain unknown.

**Methods:**

To verify the different effects of microbiota from different sexes on stroke outcomes, we performed mouse fecal microbiota transplantation (FMT) and established a model of ischemic stroke. Male and female mice received either male or female microbiota through FMT. Ischemic stroke was triggered by MCAO (middle cerebral artery occlusion), and sham surgery served as a control. Over the next few weeks, the mice underwent neurological evaluation and metabolite and inflammatory level detection, and we collected fecal samples for 16S ribosomal RNA analysis.

**Results:**

We found that when the female mice were not treated with FMT, the microbiota (especially the Firmicutes-to-Bacteroidetes ratio) and the levels of three main metabolites tended to resemble those of male mice after experimental stroke, indicating that stroke can induce an ecological imbalance in the biological community. Through intragastric administration, the gut microbiota of male and female mice was altered to resemble that of the other sex. In general, in female mice after MCAO, the survival rate was increased, the infarct area was reduced, behavioral test performance was improved, the release of beneficial metabolites was promoted and the level of inflammation was mitigated. In contrast, mice that received male microbiota were much more hampered in terms of protection against brain damage and the recovery of neurological function.

**Conclusion:**

A female-like biological community reduces the level of systemic proinflammatory cytokines after ischemic stroke. Poor stroke outcomes can be positively modulated following supplementation with female gut microbiota.

## Introduction

A growing body of experimental and clinical evidence demonstrates that the brain and the gut and its microbiota are connected by a neuronal network with strong two-way interactions [1]. This system, defined as the “gut–brain axis” or the “microbiota–gut–brain axis”, is based on neural, endocrine, immunological and metabolic pathways [2]. Post-stroke, the brain sends a top-down signal directed to the intestine, and the hypothalamic–pituitary–adrenal (HPA) axis, which is activated by the integrated reactions of specific centers in the central nervous system (CNS) and the autonomic nervous system (ANS), directly or indirectly controls intestinal tract activity through the enteric nervous system, resulting in changes in intestinal motility and permeability. Subsequently, symbiotic bacteria are dominated by conditional pathogens, which translocate from the intestinal lumen to the lamina propria and even diffuse into the peripheral tissue [3]. At this point, the intestines enter a state of dysbiosis. The dysfunctional intestinal tract will also adversely worsen the outcomes of stroke. Neurohormones and neuroactive compounds released by the intestinal epithelium mediate the activation of the vagus nerve (VN) to initiate bottom-up signaling [4]. In addition, immunogenic endotoxins from the microbiota, such as lipopolysaccharide (LPS), can induce neuroinflammation directly or by activating peripheral immune cells. Some metabolites produced or stimulated by intestinal bacteria also interact with the host's immune system through the circulation, travel to the brain and regulate the function of neurons, the maturation of microglia and the integrity of the blood‒brain barrier [3].

However, whether the gut–brain axis is affected by sex is unknown. According to previous data, stroke displays some sexually dimorphic attributes [5]. After adjusting for age and prestroke functional status, as well as stroke severity, men had higher overall mortality than women. This phenomenon is found not only in human beings, but also in the experimental environment. The results of several large research studies indicated that female animals are less prone to ischemic stroke and suffer less brain damage than age-matched males, which may be related to the protective effect of sex hormones, as aggravated brain injury occurs in ovariectomized and aged women [6–8]. These findings are noteworthy given that sex alone influences the gut microbiota. In men, stroke leads to greater irreversible changes in intestinal permeability and microbial diversity, accompanied by a more severe reduction in butyric acid, a short-chain fatty acid (SCFA) that improves intestinal barrier function. Furthermore, the change in fecal microbiota diversity in males lasts for approximately 14 days, while changes normalize by day 7 in females [9]. In general, the findings of these studies suggested that sex-associated intestinal dysbacteriosis may be a key contributor to the higher prevalence and worse prognosis of acute ischemic stroke in males.

During experimental stroke, altering the composition of the gut microbiota through FMT or intragastric administration of antibiotics can significantly affect the outcomes. However, the underlying mechanism is not clear. If it is effective to replace the intestinal microbiota with that of a more dominant group from a sex perspective, stroke patients who lack a healthy gut microbiome may benefit from this treatment. Therefore, we exchanged the microbiota in female and male mice and simulated the occurrence of stroke. The neurological impairment, survival and systemic inflammation of mice were observed, and the levels of three main compounds, trimethylamine oxide (TMAO), SCFAs and tryptophan (Trp), were evaluated to demonstrate that the female microbiota is beneficial for the outcomes of experimental stroke.

## Materials and methods

### Experimental animals and groups

Male and female C57BL/6J mice (10–12 weeks, 20–25 g) were purchased from Hubei Provincial Centers for Disease Control and Prevention and were raised in the animal experiment center of Renmin Hospital of Wuhan University with a set temperature (22 °C ± 2 °C) and humidity (65 ± 5%), a 12/12-h light/dark cycle, and free access to food and water for 1 week before the experiment. All mice were treated according to the protocols approved by the guidelines for ethical review of the welfare of experimental animals. The experiment included 4 groups: male (group MM) or female (group FF) mice who received a fecal microbiota transplant from mice of the same sex, male mice that received a fecal microbiota transplant from female mice (group MF), and female mice that received a fecal microbiota transplant from male mice (group FM). After gavage, the gut microbiota was retested to ensure successful transplantation.

### Fecal microbiota transplantation [10, 11]

Fecal samples were collected from 10 male and female donor mice, diluted in PBS (120 mg feces/1 ml PBS) until they were mushy, and centrifuged at 1000×*g* for 10 min, collected the supernatant and centrifuged again at 800×*g* for 5 min. The resulting supernatant was removed, and resuspended to prepare a fecal bacterial suspension. The recipient mice were pretreated with 500 mg/mL streptomycin sulfate sterile water for 5 days to eliminate the gut microbiota, and then the fecal bacteria suspension (50 μL) was transplanted into the intestine of mice by gavage once a day for 15 consecutive days.

### Fecal collection and 16S rRNA sequencing [12–14]

The 16S rRNA gene is commonly found in bacteria and is located in the small ribosomal subunit in the bacterial genome, with a length of approximately 1542 bp. The molecular size of the 16S rRNA gene is moderate, and its mutation rate is small; thus, the 16S rRNA gene is commonly used as a vital marker in bacterial taxonomy. The sequence contains 10 conserved regions and 9 hypervariable regions. Among them, the conserved regions can be used to design primers to amplify the target fragments, and the bacterial species can be identified through the analysis of hypervariable regions. However, due to the easy degradation of RNA, 16S rDNA encoding 16S rRNA is usually used as the sequencing object.

Fecal particles were collected before middle cerebral artery occlusion (MCAO) treatment, and total microbial genomic DNA was extracted using the E.Z.N.A.^®^ Soil DNA Kit (Omega Bio-Tek, Norcross, GA, USA) according to the manufacturer’s instructions. The purity and concentration of DNA were evaluated by 1.0% agarose gel electrophoresis and NanoDrop^®^ ND-2000 spectrophotometer (Thermo Scientific Inc., USA). According to the primer pairs 338F (5′-ACTCCTACGGGAGGCAGCAG-3′) and 806R (5′-GGACTACHVGGGTWTCTAAT-3′), the hypervariable region V3–V4 was amplified by an ABI GeneAmp^®^ 9700 PCR thermocycler (ABI, CA, USA), and then, the product was identified, purified and quantified. Sequencing was conducted using the Illumina MiSeq PE300/NovaSeq PE250 platform (Illumina, San Diego, USA). The cluster analysis of operational taxonomic units (OUT) was performed by RDP Classifier against the Silva v13816S rRNA gene database, and the confidence threshold is 70%. All bioinformatic analysis was completed using the Majorbio Cloud platform. The sample similarity of the microbiota was identified by principal coordinate analysis (PCoA) based on the Bray‒Curtis dissimilarity, and the distinct difference in abundance (phylum to genera) of bacteria among the groups was determined by linear discriminant analysis (LDA) effect size (LEfSe) (http://huttenhower.sph.harvard.edu/LEfSe) analysis (LDA score > 2, P < 0.05).

### Focal cerebral ischemia [15]

The MCAO model was established 10 days after the completion of FMT. Adult mice were anesthetized with 3% isoflurane. The left external carotid artery was fully exposed and ligated with a 6-0 suture line. By opening a small orifice from the distal end of the ligation point, the 6-0 monofilament was inserted into the internal carotid artery until reaching the middle cerebral artery branch. After occlusion for 60 min, the thread plug was carefully removed to restore blood flow. During the operation, the temperature was maintained at 37 ± 0.5 °C, and the vital signs of the mice were monitored closely. The sham group underwent surgery without thread plug insertion.

### Behavioral testing [16, 17]

Behavioral testing was conducted 72 h after MCAO. Before testing, all mice were placed in the test room for 1 h to adapt to the environment. The mice were pretested before the formal experiment to acquire a baseline score. Mice that died during the experiment were excluded to avoid any deviation in the analysis of infarct volume. Animal mortality is shown as a survival curve in Fig. 4B.

*Neurological deficit scoring*. The Logan score was selected as the reference standard and was categorized as follows: 0 = no deficit; 1 = forelimb weakness and body turning to the ipsilateral side when held by the tail; 2 = circling to the ipsilateral side; 3 = unable to support the weight on the affected side; and 4 = no spontaneous activity or barrel rolling.

*Rotarod test*. To assess motor function, the mice were placed on a rotating rod, and three consecutive tests were performed after a routine training session, with a 30-min rest between each test. The average riding time of the mice on the rotating rod in the three trials was used for analysis.

### Infarction volume measurement [16]

After deep anesthesia with isoflurane, the mice were euthanized, and their brain tissues were obtained, cut into 2 mm slices, soaked in 2,3,5-triphenyltetrazolium chloride (TTC, G1017, Servicebio, Wuhan, China) for approximately 20 min and fixed with 4% paraformaldehyde overnight. NIH ImageJ software (NIH, Bethesda, MA, USA) was used to measure the cerebral cortex area and infarct area. The percentage of the infarct volume in the contralateral cerebral cortex was taken as the end result after correction for cerebral edema.

### Isolation of brain mononuclear cells and flow cytometry analysis [18]

Three days after reperfusion, the mice were deeply anesthetized by excess isoflurane and perfused with pre-cooled physiological saline, and the ischemic ipsilateral brain tissues of each group were collected in FACS buffer (PBS with 1% fetal bovine serum), homogenized and filtered, and resuspended with 14 ml FACS buffer and 6 ml of 90% Percoll (17089101, Gelifesciences, Sweden). Then, 2 ml of 70% Percoll was slowly added to the cell suspension, which was then centrifuged at 2470*g* and 4 °C for 30 min. The intermediate layer cells were collected, washed with FACS buffer, and labeled with an appropriate amount of CD45 (11-0451-85, eBioscience, USA), CD11b (45-0112-82, eBioscience, United States), CD3 (11-0032-82, eBioscience, USA) and B220 (12-0452-82, eBioscience, USA) antibodies under dark conditions for 30 min. Sample data obtained on a Cyto FLEX flow cytometer (C02945, Beckman Coulter, USA) were analyzed by FlowJo V10 software (v10.0.7, Tree Star, Ashland, OR, USA).

### Confocal immunofluorescence staining [16, 19]

Mice were euthanized in the same way as before and perfused with pre-cooled saline, followed by 4% paraformaldehyde. After fixation for 3 days, the brains were sliced into coronal sections of 40 μm, incubated with 5% bovine serum albumin (BSA) for 1 h, and then incubated for 24 h at 4 °C with anti-CD68 (MCA1957, AbD Serotec, United Kingdom) and anti-MPO (60130, STEMCELL Technologies, Canada), both diluted to 1:200. Sections were rinsed 3 times, incubated at room temperature for 2 h with Alexa Fluor 488-conjugated antibody (ANT023, Antgene, China) for CD68 and Alexa Fluor 594-conjugated antibody (ANT030, Antgene, China) for MPO, both diluted to 1:400; washed; and then stained with 4′,6-diamidino-2-phenylindole (DAPI). Images were obtained with an Olympus laser confocal microscope. To determine the number of CD68- and MPO-reactive cells, three randomly chosen slides (0.20–0.70 mm rostral to the bregma) from each mouse were processed with ImageJ software (NIH) to count the number of cells and calculate the mean. All outcomes were assessed by two different blinded investigators.

### Serum collection

The mouse’s neck was tightly grasped with the left thumb and index finger and pressed on both sides to engorge the retro-orbital venous plexus, which was pierced at a 45-degree angle from the right or left eye with a glass capillary, and the penetration depth was 2–3 mm. As the blood flowed into the capillary tube naturally, the capillary tube was pulled away, and a dry cotton ball was used to apply direct pressure for hemostasis. The obtained blood was transferred to a centrifuge tube and placed for 30 min at room temperature for the collection of the upper serum. The levels of TMAO and Trp were measured by ELISA kits (purchased from Shanghai Yubo Life Technology Corporation, CHN) according to the manufacturer’s instructions. Changes in the levels of inflammatory cytokines in the peripheral circulation were examined by Cytometric Bead Array (CBA) kits (BD Biosciences, #560485) and analyzed by FCAP Array (BD Biosciences, USA).

### Statistical analysis

All the data are expressed as the mean ± standard deviation (SD). Student’s t tests and 2-way analyses of variance (ANOVAs) were performed to assess multiple comparisons, followed by post-tests. When ANOVAs showed significant differences, Bonferroni’s tests were used in pairwise comparisons between groups. *P* < 0.05 was considered statistically significant.

## Results

### Gut microbiota population difference between female and male mice

**Fig. 1:**
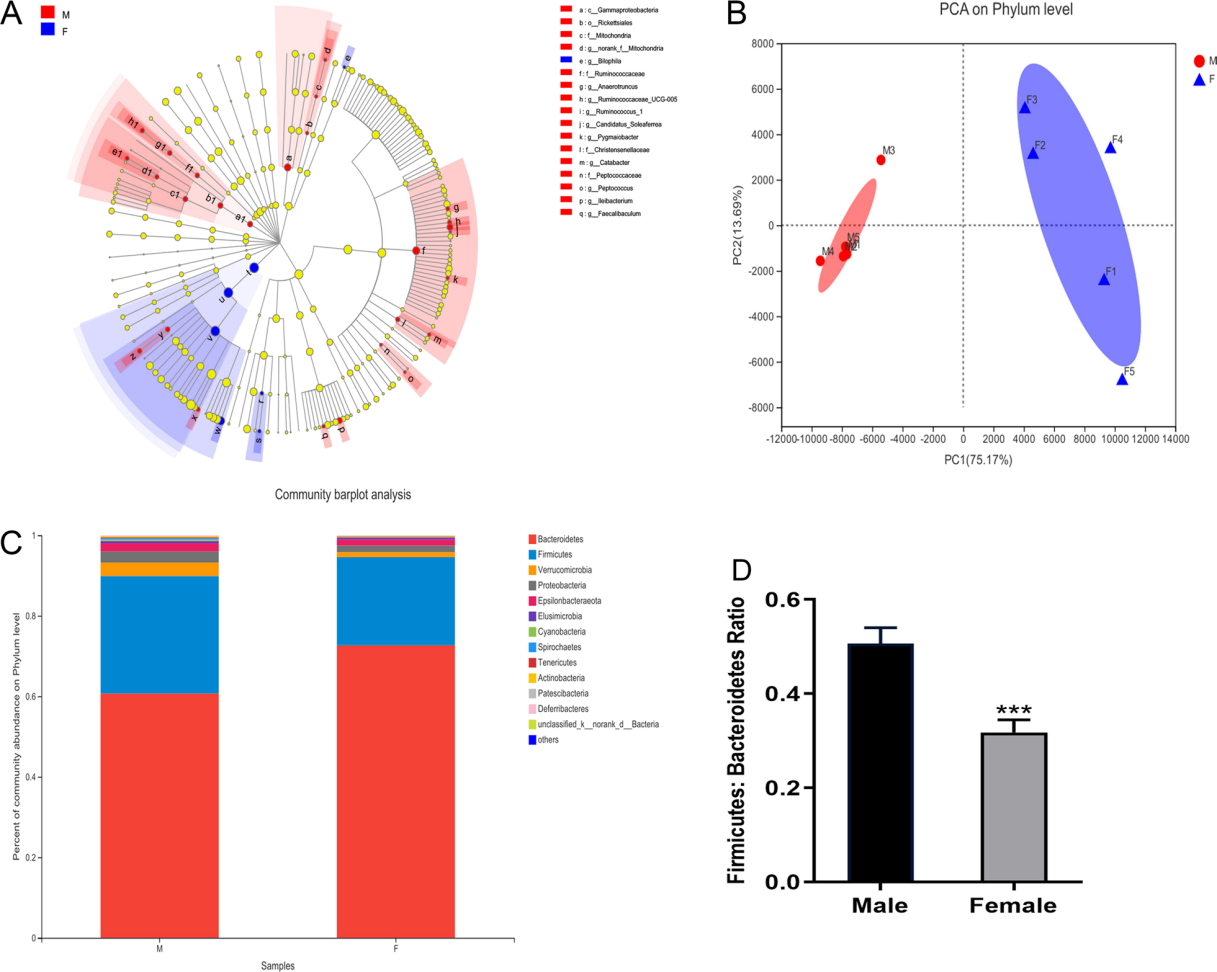
Gut microbiota population difference between female and male mice. **A** Classification of gut microbiota. **B** The PCA at the phylum level in female and male mice. **C** The composition of the microbiota in female and male mice. **D** The ratio of *Firmicutes-*to-*Bacteroidetes* in female and male mice at the phylum level. **D** The PCA at the phylum level in female and male mice. Mean ± SD. n = 5. *p: comparison between male and female mice. *p < 0.05. **p < 0.01.***p < 0.001. *PCA* principal component analysis

We first defined whether there were differences in the intestinal microbiota in the feces of male and female mice (8–12 weeks). By using 16S rRNA sequencing, LEfSe analysis suggested that the composition of the microbiota in mice differed by sex, as shown in the cladogram (Fig. 1A). Principal component analysis (PCA) further emphasized this difference. There was a statistically significant distance between the red dot, representing the male sample, and the blue dot, representing the female sample (Fig. 1B). Moreover, in the calculation of the relative abundance of microbiota in Fig. 1C, it was found that more than 90% of the bacteria were members of the phyla Firmicutes and Bacteroidetes, while the total number of the remaining bacteria was less than 10%. It is worth noting that the abundance of Bacteroidetes in males was lower than that in females, and the ratio of Firmicutes/Bacteroidetes in male mice was also significantly higher, indicating the alteration of the gut microbiota by sex. Here, we also used the F/B ratio to evaluate microbiota dysbiosis. An increase in the F/B ratio indicates a harmful imbalance [20]. Both Rhizoma coptidis and berberine have been confirmed to have an anti-obesity effect to prevent cardiovascular disease by inhibiting the ratio of Firmicutes/Bacteroidetes [21]. As shown in Fig. 1D, the F/B ratio in male mice was significantly higher than that in female mice (**p < 0.01 for the comparison between male and female mice).

### Microbiota alteration after MCAO and fecal transplantation

**Fig. 2:**
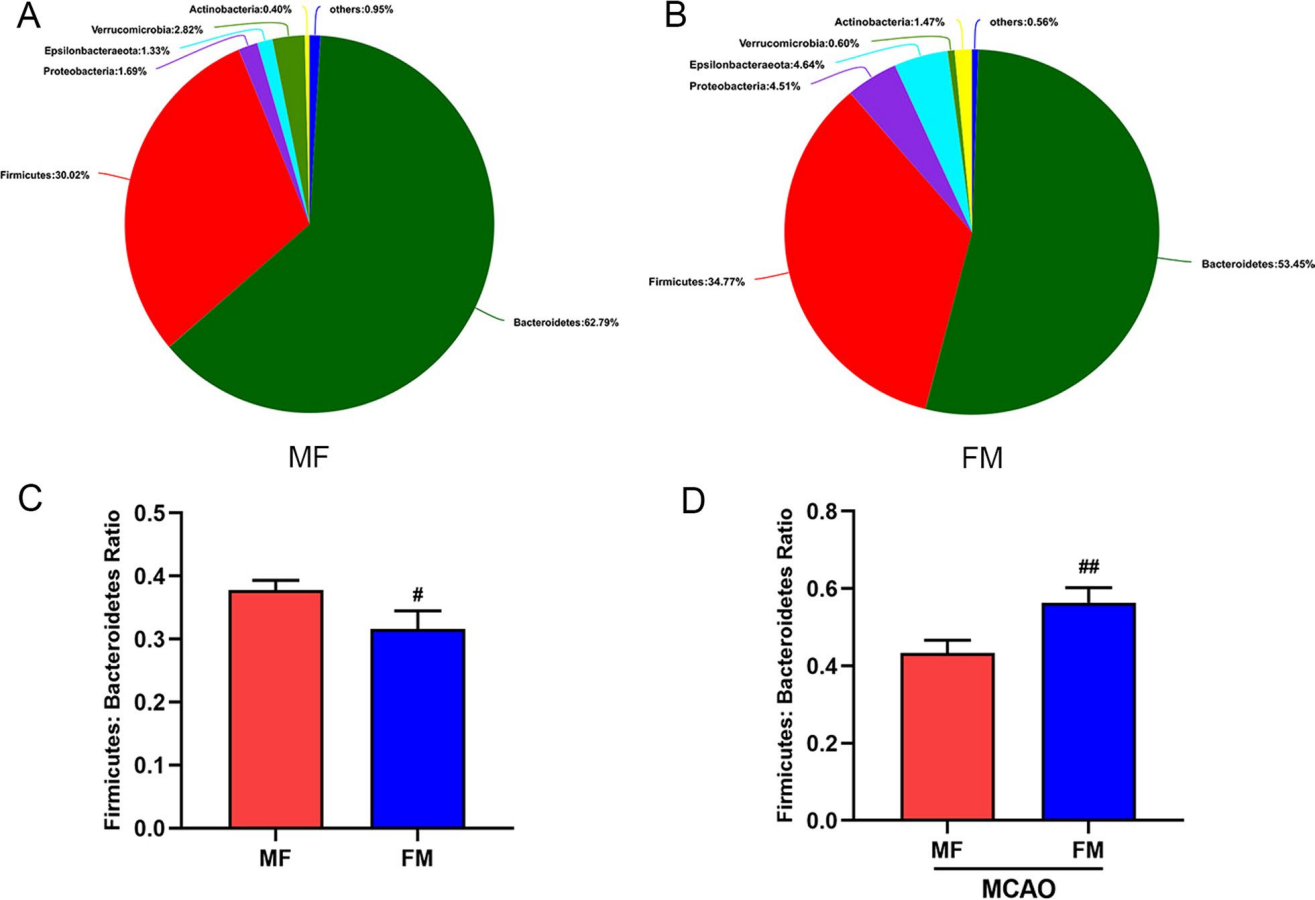
Microbiota alteration by MCAO and fecal transplantation. **A**, **B** Pie chart of phyla in the MF and FM microbiota in feces. **C** Firmicutes/Bacteroidetes (F:B) ratio in female and male mice receiving donor microbiota. **D** Firmicutes/Bacteroidetes (F:B) ratio in the MF and FM groups post MCAO. Mean ± SD. n = 5. ^#^*p*: comparison between recipient mice harboring different-sex microbiota. ^#^*p* < 0.05, ^##^*p* < 0.01, ^###^*p* < 0.001. *MCAO* middle cerebral artery occlusion

According to previous studies [1, 11], after antibiotic sterilization, intragastric administration of a collected fecal suspension to a recipient can transmit some donor characteristics to the recipient. In this study, we also adopted this method and verified the effect of FMT through gut microbiota composition analysis. This analysis mainly included the MF group, in which male mice were transplanted with female microbiota, and the FM group, in which female mice were transplanted with male microbiota (Fig. 2A, B). As before, the F/B ratio was still our focus. After 7 days of continuous intragastric administration, the situation was reversed. Compared with recipient mice, MF mice showed a significantly lower F/B ratio, while FM mice showed a higher F/B ratio. Two-way ANOVA showed significant effects on recipients (Fig. 2C, ^##^*p* < 0.001), which indicated that our transplantation was successful.

Subsequently, we assessed the relative abundance of the mouse gut microbiota 60 min after MCAO. As described in Fig. 2D, MCAO increased the proportion of Firmicutes in the flora and decreased the proportion of Bacteroidetes. Compared with the data in Fig. 2C, the F/B ratio increased by approximately 26% in the MF group and by 63% in the FM group 3 days post stroke. The results showed that stroke induced biological dysbiosis in mice, and the homeostasis of the FM group flora was more seriously damaged.

### Effects of altered microbiota on infarct volume, behavioral outcomes and mortality

**Fig. 3:**
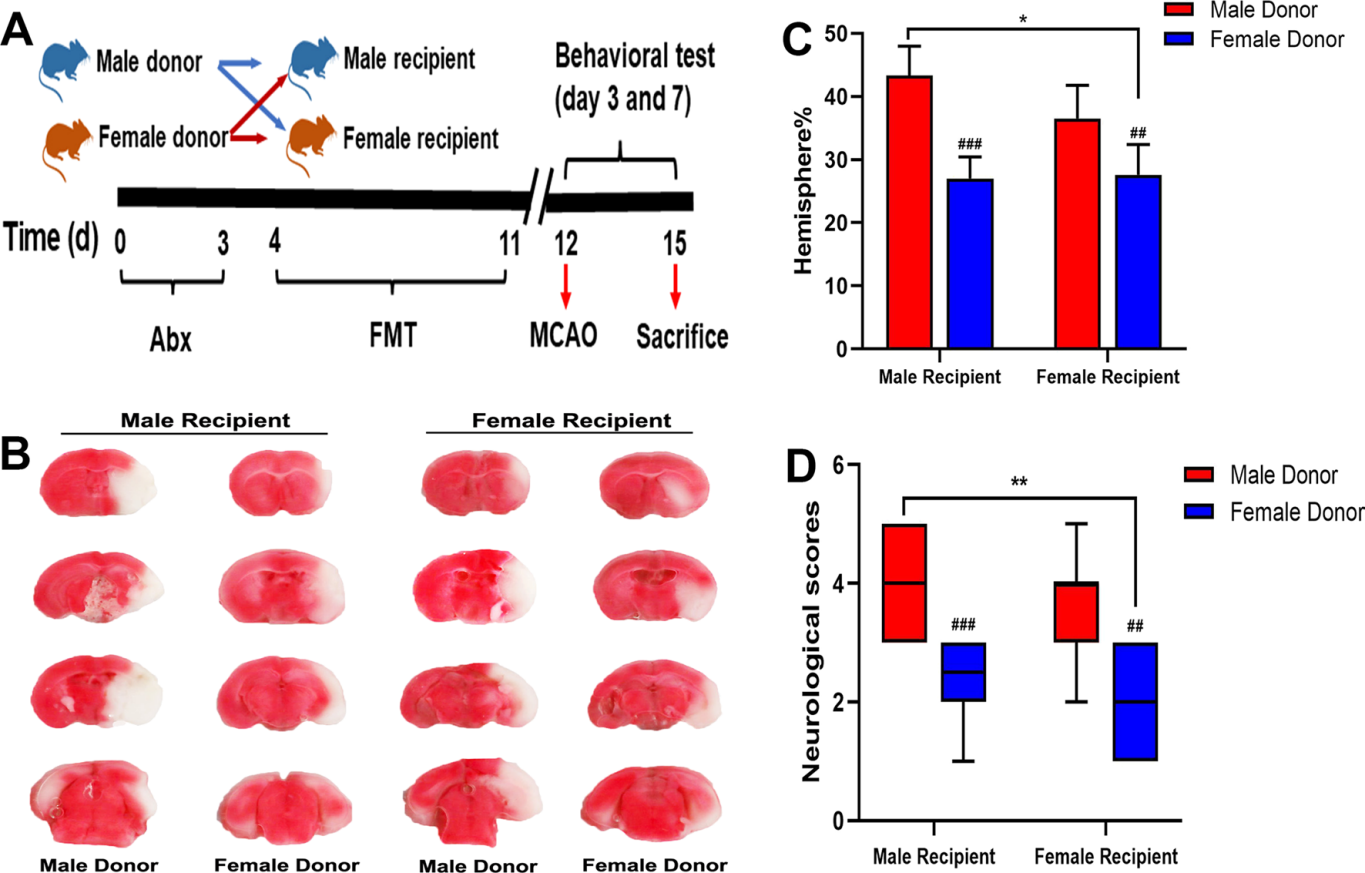
Female microbiota transplantation reduced infarct size and cerebral injury after MCAO. **A** The experimental protocol for fecal microbiota transplantation. **B** TTC staining was used to measure the post-stroke infarct volume of the mice in each group. **C** The infarct volume of each group was quantitatively analyzed and expressed as a percentage. **D** Scores of neurological deficits in each group. Mean + SD. n = 10 each group. ^#^*p*: comparison between recipient mice of the same sex. ^#^*p* < 0.05, ^##^*p* < 0.01, ^###^*p* < 0.001. *p: comparison between recipient mice harboring the same-sex microbiota. *p < 0.05. **p < 0.01. ***p < 0.001

To clarify the effect of the intestinal microbiota, the mice implanted with FMT were killed 3 days after MCAO, and the physiological changes in vivo were detected. After the brain slices were collected, they were stained with TTC staining solution, and the white area represented the ischemic infarction (Fig. 3B). Statistical analysis found that in male recipients, female microbiota reduced the infarct volume by approximately 15%, and in female recipients, male microbiota expanded the infarct range by 10%. Therefore, regardless of sex, female microbiota had a significant protective effect (Fig. 3C). The Longa score is a commonly used method for scoring neurological function in animals, where higher scores indicate more severe nerve defects. In male recipients, male donors and female donors had average scores of 4 and 2.4, respectively, while in female recipients, the scores were 3.6 and 2.1 (Fig. 3D).

**Fig. 4:**
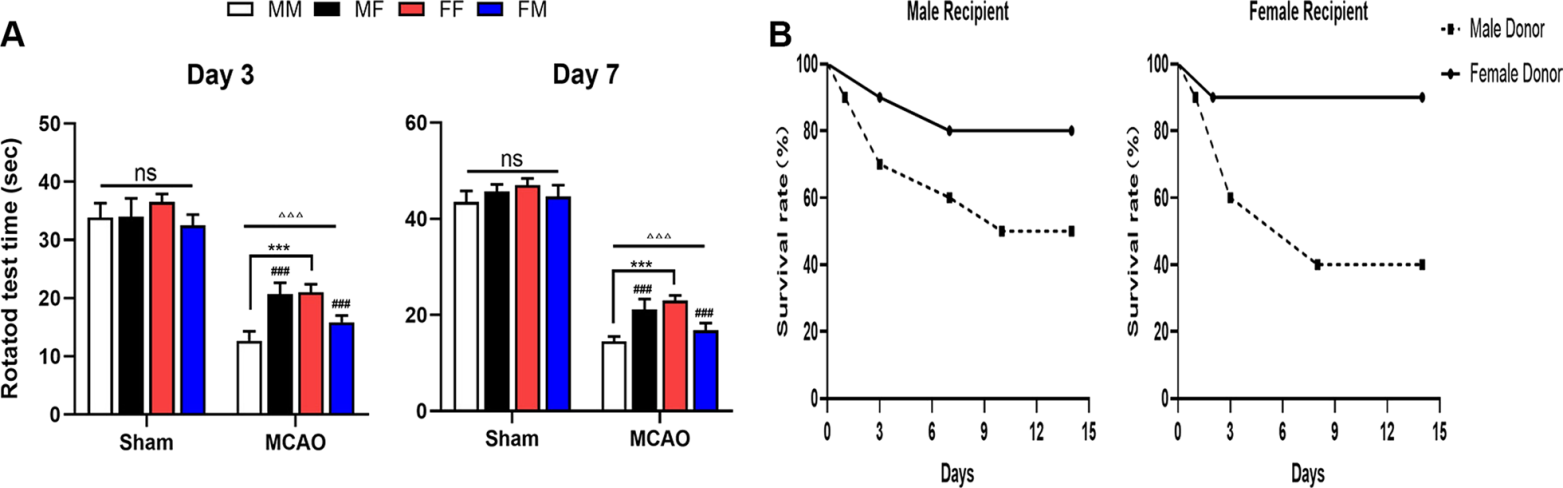
Impact of gut microbiota on behavioral outcomes and mortality. **A** The behavior of mice in each group was detected in the rotarod experiment. **B** Changes in the survival curve of mice in each group after MCAO. ^△^*p*: MCAO vs. sham. ^△^*p* < 0.05. ^△△^*p* < 0.01. ^△△△^*p* < 0.001. ^#^*p*: comparison between recipient mice of the same sex. ^#^*p* < 0.05, ^##^*p* < 0.01, ^###^*p* < 0.001. *p: comparison between recipient mice harboring same-sex microbiota. *p < 0.05. **p < 0.01. ***p < 0.001

The longer-term effects of FMT are reflected by the results of behavioral experiments and survival monitoring. There was no remarkable difference between the sham group, however, at 3 days after MCAO, the rotation time of the MM group and FM group was shorter than that of the FF group and MF group, and this phenomenon persisted in the recovery period, that is, 7 days after MCAO. This finding indicates that the effect of FMT was persistent, but it should be noted that at this time point, the rotation time of all mice was prolonged, indicating that neurological function gradually improved (Fig. 4A). The same is true for the time of survival. We monitored survival for 14 days after stroke, and overall, fewer mice died when they were transplanted with female microbiota (Fig. 4B).

### Impact of mouse gut microbiota on metabolites

**Fig. 5:**
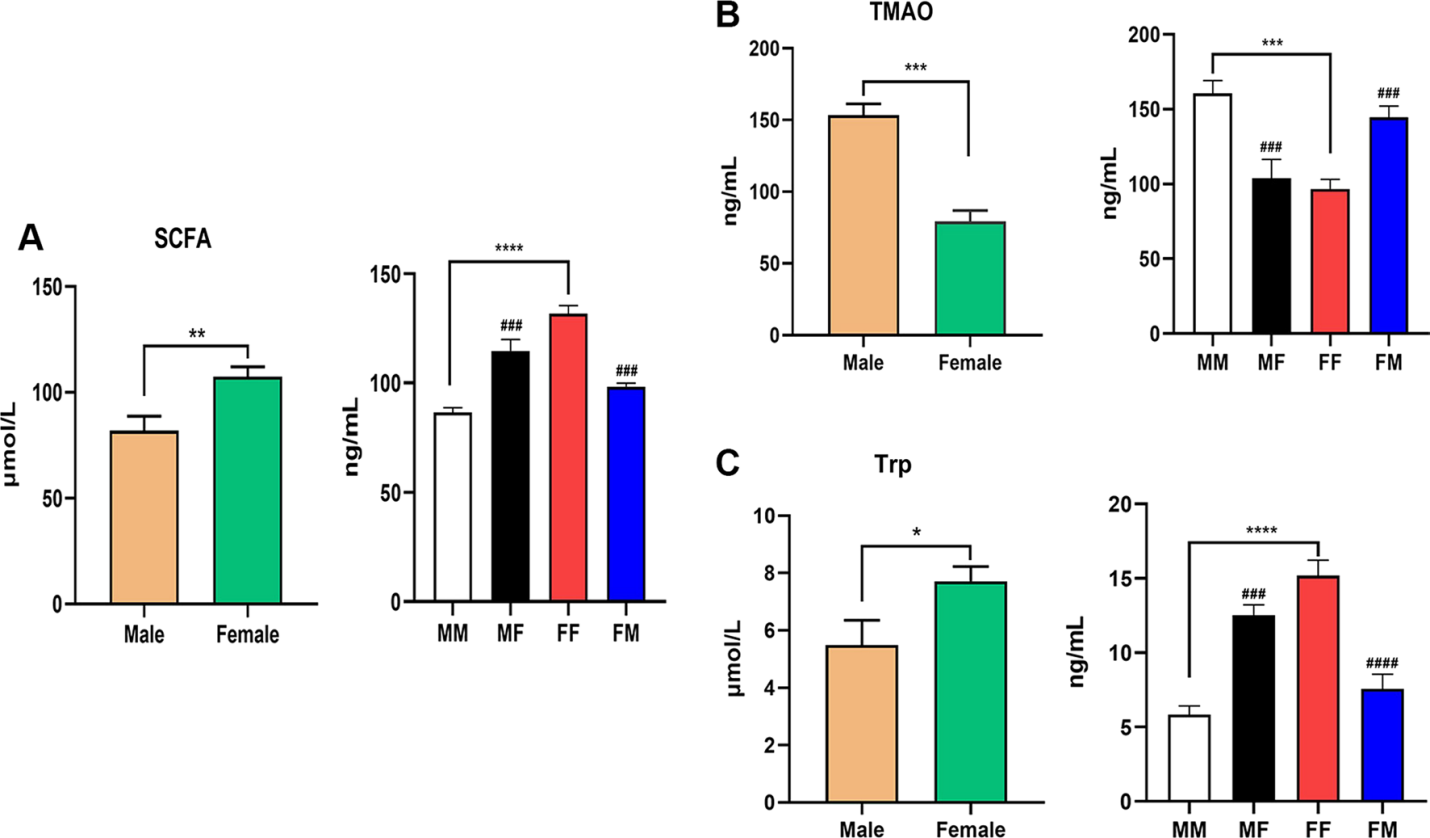
Fecal microbiota transplantation altered gut microbiota metabolites in the serum of male and female mice 72 h after cerebral infarction. **A** Serum SCFA concentration in different groups under normal conditions and after transplantation. **B** Serum TMAO concentration in each group under normal conditions and after transplantation. **C** Serum Trp concentration in each group under normal conditions and after transplantation. Mean ± SD. n = 8. *p: comparison between males and females. *p < 0.05. **p < 0.01. ***p < 0.001. ^#^*p*: comparison between recipient mice of the same sex. ^#^*p* < 0.05, ^##^*p* < 0.01, ^###^*p* < 0.001. *SCFAs* short-chain fatty acids, *TMAO* trimethylamine oxide, *Trp* tryptophan

Given the important role of TMAO, Trp and SCFAs in the intestinal-microbiota–brain axis [22, 23], we also detected these common metabolites. It is known that these metabolites are involved in the pathogenesis of ischemic stroke; TMAO aggravates stroke injury, while Trp and SCFAs are protective [1, 24, 25]. The left side of each part of Fig. 5 represents mice that did not undergo FMT or MCAO, and the figure shows that males had higher levels of TMAO and lower levels of SCFAs and Trp than females. The right side represents the mice with fecal bacteria intragastric administration and 1 h of MCAO. 72 h after stroke, the serum SCFA concentration was 86.54 ng/ml in the MM group, 114.7 ng/ml in the MF group, 98.29 ng/ml in the FM group and 131.85 ng/ml in the FF group (Fig. 5A); the serum TMAO concentration was 160.59 ng/ml in the MM group, 103.73 ng/ml in the MF group, 144.6 ng/ml in the FM group and 96.67 ng/ml in the FF group (Fig. 5B); and the serum Trp concentration was 5.85 ng/ml in the MM group, 12.54 ng/ml in the MF group, 7.58 ng/ml in the FM group and 15.19 ng/ml in the FF group (Fig. 5C). In light of the properties of these three metabolites, these results were consistent with the neurological function of the mice.

### Reduction of inflammatory cells infiltration in ischemic brain tissue in mice harboring female microbiota after MCAO

**Fig. 6:**
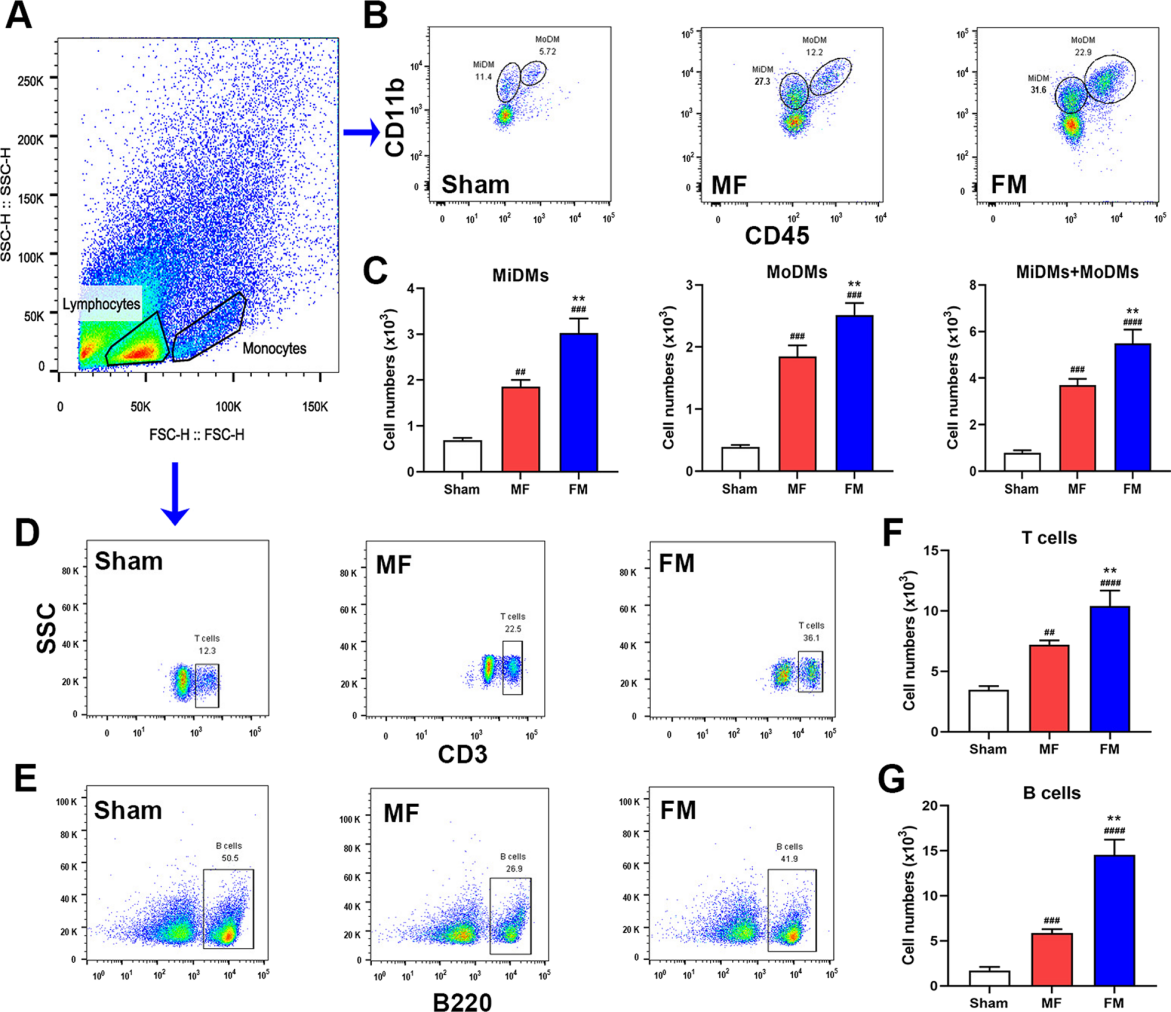
Reduced inflammatory cells in ischemic brain tissue in mice harboring female microbiota. **A**, **B** Gating strategies for monocytes and macrophages derived from microglia (CD11b + CD45inter) and macrophages derived from monocytes (CD11b + CD45high). **C** The infiltration of microglia and macrophages. **A**, **D**, **E** Gating strategies for T lymphocytes (CD3^+^) and B lymphocytes (B220^+^). **F**, **G** The infiltration of T and B lymphocytes in the infarcted hemisphere. Mean ± SD. n = 8–12/group. ^#^*p*: MCAO vs. sham. ^#^*p* < 0.05. ^##^*p* < 0.01. ^###^*p* < 0.001. *p: comparison between recipient mice receiving fecal microbiota from different sexes. *p < 0.05, **p < 0.01, ***p < 0.001

On the third day after the MCAO operation, we extracted the tissue from the infarcted area of the brain to make a mononuclear cell suspension, and we counted the number of microglia (CD45^+^CD11b^inter^), peripheral macrophages (CD45^+^CD11b^hi^), T cells (CD3^+^) and B cells (B220^+^) by flow cytometry (Fig. 6). The results showed that compared with the sham group, the aggregation of various immune cells in the ischemic area increased after MCAO, but the difference in the MCAO group was that the infiltration of microglia, macrophages, T cells and B cells in the FM group with male microbiota was significantly higher than that in the MF group with female microbiota.

### Microglia/macrophage and neutrophil infiltration post-stroke after gut microbiota transplantation

**Fig. 7:**
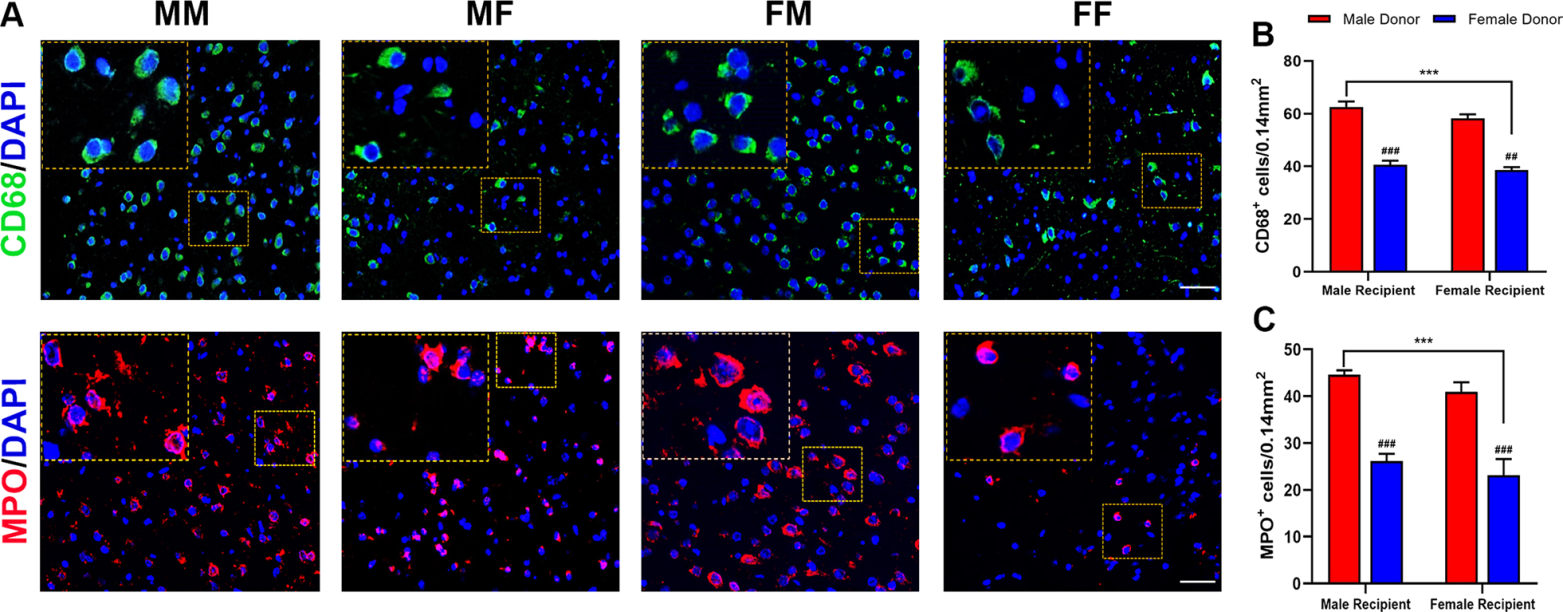
Fecal microbiota transplantation reduced the quantity of CD68- and MPO-positive immune cells in the ischemic penumbra. **A** Representative immunofluorescence images of CD68- and MPO-stained brains counterstained with DAPI. **B** Number of CD68 + cells per unit area under confocal microscopy. **C** Number of MPO + cells per unit area under confocal microscopy. Mean ± SD. n = 5–7/group. Scale bar = 30 µm. ^#^*p*: comparison between recipient mice of the same sex. ^#^*p* < 0.05, ^##^*p* < 0.01, ^###^*p* < 0.001. *p: comparison between recipient mice harboring the same-sex microbiota. *p < 0.05. **p < 0.01. ***p < 0.001

In addition, we used an immunofluorescence method to label microglia/macrophages and neutrophils in the cerebral ischemic area to detect their infiltration and distribution. This method illustrates the effect of the intestinal microbiota on brain inflammation after ischemic stroke (Fig. 7A). According to the data on the number of CD68− and MPO-immunoreactive cells, we found that 72 h after ischemia, CD68^+^ microglia/macrophages and MPO^+^ neutrophils were observed in the infarcted area, but the infiltration of these two kinds of cells was significantly inhibited in the two groups of mice transplanted with female microbiota, while the opposite was true in the mice transplanted with male microbiota (Fig. 7B).

### Influence of FMT on the mRNA expression level of inflammatory factors in infarcted brain tissue 72 h after MCAO

**Fig. 8:**
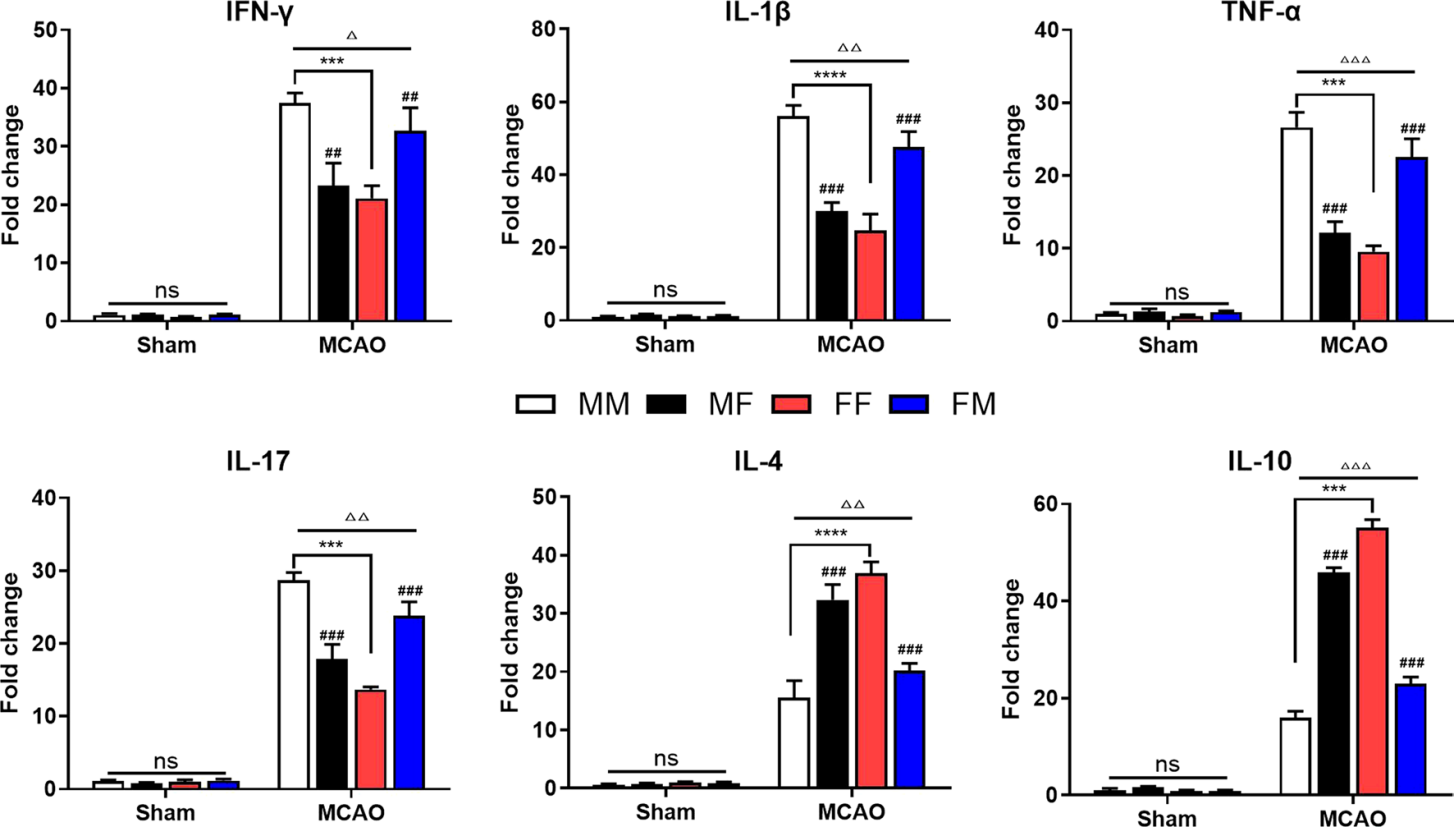
Fecal microbiota transplantation changed the mRNA expression levels of inflammatory factors after ischemic stroke. The total RNA of the 4 groups was extracted from infarcted regions 3 days after MCAO. The gene expression levels were normalized to those of GAPDH. Mean ± SD. n = 5–7/group. ^△^*p*: MCAO vs. sham. ^△^*p* < 0.05. ^△△^*p* < 0.01. ^△△△^*p* < 0.001. ^#^*p*: comparison between recipient mice of the same sex. ^#^*p* < 0.05, ^##^*p* < 0.01, ^###^*p* < 0.001. *p: comparison between recipient mice harboring the same-sex microbiota. *p < 0.05. **p < 0.01. ***p < 0.001

To further observe the effect of FMT on the inflammatory response, the expression of the proinflammatory factors IFN-γ, IL-1β, IL-17, and TNF-α and the anti-inflammatory factors IL-4 and IL-10 in the cerebral infarct area was detected by RT‒PCR. The IFN-γ, IL-1 β, IL-17, and TNF-α were significantly higher in the MM group, which was reversed by FMT. In female mice, the basal levels of these proinflammatory factors were lower, but they did not seem to be affected by gut microbiota transplants from male mice. However, the IL-4 and IL-10 had a trend that was opposite that of the proinflammatory cytokines. In summary, the intestinal microbiota of female mice could antagonize the development of inflammation (Fig. 8).

### Attenuation of circulating inflammatory factors 72 h after MCAO by female microbiota

**Fig. 9:**
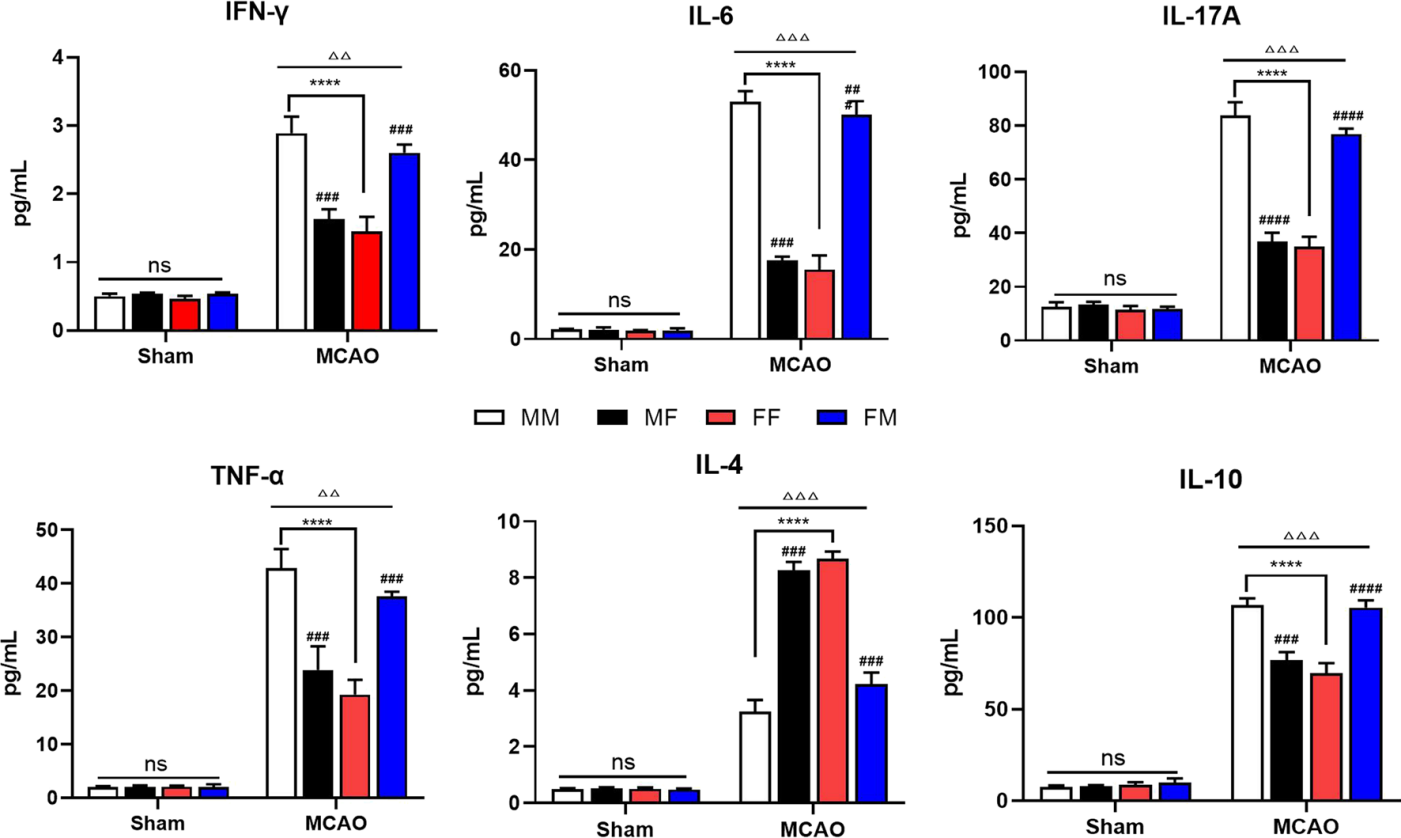
Fecal microbiota transplantation inhibited the secretion of inflammatory factors in the serum 72 h after MCAO. CBA kit analysis of the concentrations of IFN-γ, IL-6, IL-17a, TNF-α, IL-4 and IL-10 in the serum of each group 72 h after MCAO. Mean ± SD. n = 8–10/group. ^△^*p*: MCAO vs. sham. ^△^*p* < 0.05. ^△△^*p* < 0.01. ^△△△^*p* < 0.001. ^#^*p*: comparison between recipient mice of the same sex. ^#^*p* < 0.05, ^##^*p* < 0.01, ^###^*p* < 0.001. *p: comparison between recipient mice harboring same-sex microbiota. *p < 0.05. **p < 0.01. ***p < 0.001

The immune pathway is one of the ways that the intestinal flora affects the outcomes of stroke. Both central inflammation and peripheral inflammation may be affected by FMT to some extent. To confirm this hypothesis, we extracted peripheral blood from mice in each group, and we used a CBA kit to detect changes in IFN-γ, IL-6, IL-17a, TNF-α, IL-4 and IL-10 in serum. The results demonstrated that the protective effect of female microbiota was also reflected in the peripheral blood, manifesting as predominant levels of IL-4 and IL-10. FMT reduced the burden of the massive increase in proinflammatory factors (IFN-γ, IL-6, IL-17a, and TNF-α) in the MF group after MCAO, but the predominant level of anti-inflammatory factors (IL-4 and IL-10) in the FM group was still negligible after FMT (Fig. 9).

## Discussion

The findings of this study show that the microbiota of male and female mice has a different composition and thus different effects. Without any treatment, there was a higher baseline F/B ratio in male mice. A higher abundant Firmicutes and a lower abundant of Bacteroidetes are often manifestations of serious diseases [26]. In the experimental stroke model established in male mice, female microbiota transplantation transmitted the protective characteristics of females to males. Brain damage, especially the reduction in systemic inflammation and the improvement in prognosis, could be observed. In contrast, the microbiota from male mice aggravated the adverse consequences. These results suggest a link between sex differences in the intestinal microbiota and ischemic stroke, and regulating the components of intestinal microbiota may provide a promising therapeutic strategy.

The sex difference in ischemic stroke is an important feature in stroke epidemiology and has a considerably profound effect on stroke outcomes [27]. From neonates to adolescents, males account for a significantly higher proportion (57–63%) of the ischemic stroke incidence and increased risk of adverse outcomes [28]. This male predominance persists in ischemic stroke subtypes or stratification by other etiologies [28–30]. Clinical and experimental evidence [31, 32] shows that natural sex bias in the gut microbiota also exists, which may be due to the influence of sex hormones. Estrogen, progesterone and testosterone, are the major decisive factors of sex differences in mammals [33]. Studies have shown that sex hormones generally influence the composition of the sex-specific gut microbiota by regulating intestinal homeostasis in the following ways [34–36]: (i) direct gene expression regulation in intestinal epithelial cells by binding with specific nuclear steroid hormone receptor (SHR) transcription factors; (ii) participation in the TLR pathway to change the inflammatory immune environment; and (iii) the activity of orphan nuclear estrogen-related receptor α (ESRRA) in mitochondrial function. In agreement, α-diversity was significantly different between the sexes only among postpubescent mice [37]. Among the microbial families involved, consistently higher abundances of Veillonellaceae, Peptococcaceae and Lactobacillaceae (all of three belong to phylum Firmicutes) were observed in adult males than in adult females or castrated males [37]. Regardless of age, males harbor a higher intestinal Firmicutes/Bacteroidetes ratio than females. Firmicutes and Bacteroidetes phyla are of great importance in the human microbiome, representing Gram-positive bacteria and Gram-negative bacteria in the gastrointestinal tract, respectively. Perturbations in the proportional composition of these two taxonomic groups may provide an understanding of the host’s health status [38]. Usually, the F/B ratio increases with age and is regarded as an hallmark of gut dysbiosis, with a higher F/B ratio implying a more disordered microbiota composition [39]. Our finding is comparable to data reported in numerous studies [40–42] that concluded that sex alters the intestinal flora in mice and humans. When comparing fecal samples from male and female mice of the same age, we found a clear sex difference at the phylum level and a significantly higher F/B ratio in male mice. Coincidentally, this was generally consistent with the sex differences in ischemic stroke outcomes. Male mice with higher F/B ratios were more severely injured, manifested by a larger infarct size, higher neurobehavioral scores, and lower survival rates. Therefore, we propose that the sex-specific discrepancy in the gut microbiota provides potential mechanisms for the development or outcomes of pathological states related to sex.

Our experiments further demonstrate the credibility of this proposition through the post-stroke transplantation of fecal bacteria from females to males and from males to females. After FMT, microbiota analysis showed that male mice that received female microbiota also obtained a female-specific low F/B ratio, and vice versa, which was consistent with the performance after MCAO. On the 3rd day post MCAO, both males and females with male microbiota had larger infarct sizes, increased neurological deficit scores, and decreased exercise times on the rotating rod (the same was true on day 7). In terms of mortality, female microbiota also had beneficial effects. According to the results, the group that received female microbiota obtained an advantage. Not only did 90% of the females survive, but their mortality rate was approximately 30% lower than that of the males. Considering all data, at the same age, male microbiota was a detrimental factor and negatively impacted outcomes in either the acute or the chronic phase of MCAO compared with female microbiota. The determination of these specific factors is an important step to extend the experiment to practical treatment research.

In the context of stroke, the interaction between the intestinal microbiota and the CNS is the result of two-way communication [3], which is also illustrated by our results. First, we found that MCAO further led to an imbalance in the intestinal microbiota as observed in the baseline F/B ratio in both male and female mice, with an increase in approximately 25% and 37%, respectively. The male microbiota was more severely affected. Second, the gut microbiota of the different sexes also affected the degree of stroke damage, behavioral ability, and mortality. These two findings represent "top-down" and "bottom-up" signal transduction in the gut–brain axis [43], respectively. On the whole, stroke is a sex-related disease that is also intrinsically regulated by sex-related intestinal microbiota. Sex is a factor that plays an undeniable role in the gut–microbiota–brain axis.

As mentioned earlier, signal transmission between the brain and the intestinal tract occurs through neural and nonneural mechanisms, mainly including the nervous system (mainly the ENS and the VN), the HPA axis, the immune system, and microbiota-derived compounds [3]. In terms of signal transduction, the VN and the ANS are the main methods of direct communication between the intestine and the brain [4]. Through neuron–glial–epithelial units or intestinal effector cells, regulatory information is sent to the intestinal microenvironment, affecting intestinal homeostasis and regulating intestinal barrier permeability, intestinal peristalsis and the mucosal immune response [43]. In addition, the HPA axis is also particularly important in the communication of coping with the stress response, regulating the release of glucocorticoids, saline corticosteroids or catecholamines to change the quantity and quality of intestinal microbiota, as well as neuroendocrine responses [44]. After ischemic stroke, the neural pathway is abnormally regulated, resulting in an imbalance in the intestinal microenvironment, forming a pathological intestinal microbiota, releasing a large number of harmful signals and promoting the development of the disease [4].

Signals from the intestine to the brain mainly depend on crosstalk between intestinal microbes and the other three mechanisms. This crosstalk is achieved by stimulating the hepatic and ventral branches of the VN to carry the signal to the brain, affecting the activity of neurons [4] and regulating the expression of corticotropin-releasing factor (CRF) in the hypothalamus and brain-derived neurotrophic factor (BDNF) and *N*-methyl-d-aspartic acid receptor (NMDA) receptors in the cerebral cortex and hippocampus, which are important regulators of HPA axis function [43]. The development and function execution of microglia, the in situ immune cells of the brain, are also regulated by the host microbiota [45, 46]. Microbial compounds and metabolites from intestinal flora, including indole, tryptophan, histamine, SCFAs and TMAO, are important media for communication between the intestinal tract and the brain that are thought to enter the brain through the blood circulation and trigger responses through a variety of mechanisms [47].

TMAO, SCFAs and Trp are three bacterial metabolites that have been shown to be associated with stroke [48–50]. TMAO is the product of some dietary nutrients, such as L-carnitine, phosphatidylcholine and choline, processed by intestinal microorganisms and transformed by heparin monooxygenase [51]. Large-scale clinical case‒control studies [51, 52] have shown that a high concentration of plasma TMAO is positively correlated with the aggregation of proinflammatory mediators CD14^+^/CD16^+^ monocytes and the incidence of ischemic stroke, which is regarded as a predictor of poorer functional outcome events and mortality [24]. Earlier reports also showed that the ability to produce TMA (the precursor of TMAO) is strong in Firmicutes but very weak in Bacteroidetes [53]. In contrast to TMAO, SCFAs and Trp are small molecules that symbolize the positive side. The discovery of SCFAs was of special significance to the study of the pathogenesis of stroke and rehabilitation, and experimental animal studies have confirmed the therapeutic effect of their biological characteristics in stroke [1, 49]. SCFAs activate G-protein coupled receptors to regulate the immune system and play an anti-inflammatory role [54]. In addition, known propionate producers belong to the genera Bacteroides and Prevotella [55]. The transplantation of feces rich in SCFAs (especially butyric acid) can lead to microbiota remodeling, increase lactic acid bacteria species, improve the intestinal microbiota, and thus play a positive role in regulating the cerebral ischemic response [4]. Moreover, previous studies have proven that tryptophan degrades indole derivatives, such as tryptophan, 3-indoleacetic acid (IAA), and indolepropionic acid (IPA), which bind to Ah receptors in the CNS, activate Ah signal transduction and inhibit CNS inflammation [25]. In turn, the perturbation of inflammation can regulate the metabolism of tryptophan to kynurenine (KYN) or serotonin (5-hydroxytryptamine; 5-HT) in mice exposed to stress; inflammatory cytokines such as interferon-γ (IFN-γ) and IL-6 trigger the production of indoleamine-2,3-dioxygenases (Idos) [56], which metabolize Trp to KYN, partially explaining the reduction in Trp in males. Compared with those of female mice, the SCFAs and Trp of male mice decreased by approximately 1/3 and increased by approximately 1/2, respectively, and similar changes occurred in the transplantation group, which was associated not only with the level of bacterial metabolism but also with an increased inflammatory response after MCAO and more severe nerve injury. Although many previous studies have shown the correlation and influence between these three metabolites and stroke outcomes, combined with sex, our research further confirmed these conclusions. It is necessary to conduct more in-depth research in this field. Unfortunately, we did not detect the amounts of metabolites in feces, which may make the data inaccurate and less comprehensive. Since TMAO, SCFAs and Trp are not affected by the high selectivity of the blood‒brain barrier to enter the brain through the peripheral blood circulation, we believe that the concentration in serum can also reflect the ability of bacteria to decompose food.

In the complex interaction network of the gut–microbiome–brain axis, inflammation is inevitably discussed, whether it is through top-down signal transmission or bottom-up reverse regulation. Similarly, inflammation is also a key link in the pathogenesis of ischemic stroke [57]. It has been found that an anti-inflammatory intestinal microbiota induced by antibiotic therapy in stroke mice [58] or the transplantation of anti-inflammatory donor gut microbiota into naïve mice through FMT can effectively reduce the infarct volume and maintain sensorimotor function for at least one week after MCAO [59]. More importantly, the polarization of T cells toward regulatory T cells (Tregs) was also observed in these mice, which was not conducive to the activation of inflammatory IL-17 + γδ T cells [58]. These results are consistent with our view that the intestinal microbiota of mice can regulate inflammation and affect the consequences of stroke. On this basis, we divided the intestinal microbiota into female and male microbiota and proved that compared with male microbiota, mice with female microbiota showed an increase in blood circulation and brain tissue anti-inflammatory factors and a decrease in proinflammatory factors, as well as a decrease in inflammatory cell infiltration in the infarction center after stroke. The abovementioned bacterial metabolites have also been proven to be regulatory factors of inflammation after stroke; therefore, we speculate that the changes in inflammatory cytokines in the circulation may be caused by the combined action of TMAO, SCFAs and Trp after they reach the blood circulation.

As noted, based on the background of ischemic stroke, the incidence rate and intestinal flora composition of human males, especially the Firmicutes/Bacteroides ratio, are similar to those of male mice. In our study, we found that fecal bacteria transplantation could replace the intestinal flora of males with that of females to reduce damage. At present, in some human case reports of neurological disorders, FMT, through its alteration of the Intestinal flora, has been suggested to be a promising effective therapeutic tool for diseases and conditions such as autism spectrum disorder, multiple sclerosis, Parkinson's disease, epilepsy, Tourette syndrome and diabetic neuropathy [60]. However, for ischemic stroke, only limited animal studies have been performed [60]. Hence, this study highlights the favorable prospects of FMT in the treatment of stroke; however, more studies need to be performed regarding its safety and efficacy.

Differences in sex hormone levels are primarily responsible for the sexually dimorphic microbiome. Existing studies have proven that the gut microbiota is involved in the physiological roles of sex hormones, and intestinal flora can also be regulated by hormones [61, 62]. In addition, the existence of estrogen is an important factor affecting the degree of post-stroke injury [63]; therefore, changes in this hormone may also be one of the intermediate links in the process of flora exchange. Unfortunately, this point has not been reflected in our research, which is not only a limitation of our research but also an issue that needs to be further clarified.

Based on our existing research results, there are still some problems to be solved, the first of which is how sex determines the intestinal flora composition. In addition to the widely discussed sex hormones, a sex bias has also been found in the Th17/Treg cell ratio, which is closely related to intestinal microbiota homeostasis [64, 65] and may be a potential interfering factor. Second, the specific mechanism by which dysbacteriosis affects neuroinflammation needs to be investigated. In our current study, the contents of TMAO, SCFAs and Trp in the brain were not detected, and whether they play roles directly through the blood‒brain barrier or indirectly through peripheral inflammatory factors and inflammatory cells remains to be confirmed. Third, there are other ways in which gut microbiota feedback affects stroke in addition to its metabolites. For example, outer membrane vesicles (OMVs) from intestinal bacteria are carriers for long-distance transmission of signaling molecules; these OMVs can reach the brain and release their intracellular contents to participate in the disease process [66]. It is also worth clarifying whether the components of OMVs change with sex, as well as their potential effects and mechanisms on brain tissue.

## Conclusion

**Fig. 10:**
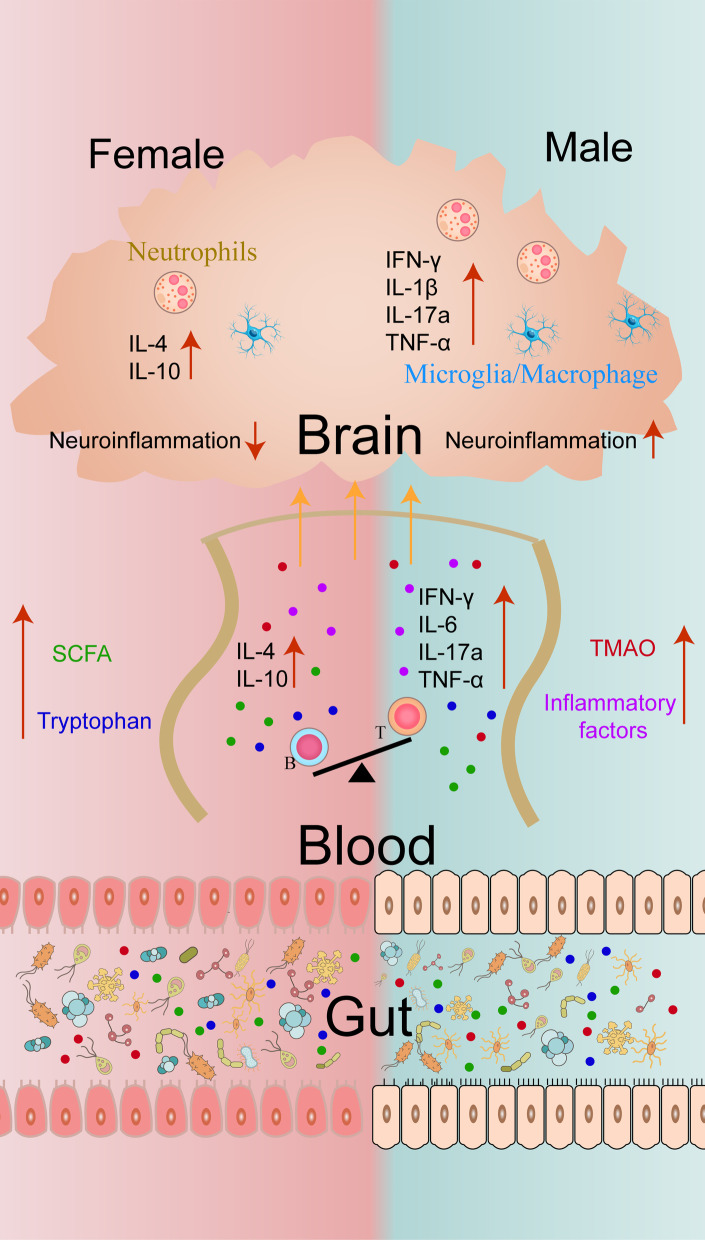
Schematic representation of the immune interaction among sex, the gut microbiome and the brain. In response to cerebral ischemia stimulation, the composition of the intestinal microbiota changes correspondingly according to sex. Bacteroides is dominant in females, secreting more SCFAs and Trp, inducing an increase in the anti-inflammatory factors IL4 and IL10 and reducing the brain aggregation of inflammatory cells such as neutrophils, microglia/macrophages, and T and B lymphocytes, thus inhibiting neuroinflammation. The male intestinal environment is conducive to the survival of Firmicutes, producing a large amount of TMAO, which promotes the production of proinflammatory factors and the infiltration of inflammatory cells in the brain, eventually aggravating central inflammation. *TMAO* trimethylamine oxide, *SCFA* short-chain fatty acid, *Trp* tryptophan

An underlying crosstalk linking the gut microbiota, sex, and immune response to the outcome of ischemic stroke was proposed in this study (Fig. 10). Biological sex shapes the composition of the gut microbiota and the subsequent intestinal metabolism and substance secretion, especially the secretion of TMAO, SCFAs and Trp, which transmit the signals of the immune reactions, including inflammatory cells and factors, triggered by the intestinal microbiota to the brain, affecting the progression and prognosis of stroke. Clinical translation would bring about a new option for the treatment of stroke. The intestinal microbiota can be artificially molded into a beneficial microbiota through diet, probiotics, antibiotics and fecal bacteria transplantation to protect against stroke or other injuries.

## Data Availability

The data obtained from this study are included in the article.

## Abbreviations

FMT: Fecal microbiota transplantation
MCAO: Middle cerebral artery occlusion
LPS: Lipopolysaccharide
HPA: Hypothalamic–pituitary–adrenal
ANS: Autonomic nervous system
TTC: 2,3,5-Triphenyltetrazolium chloride
BSA: Bovine serum albumin
TMAO: Trimethylamine oxide
SCFAs: Short-chain fatty acids
Trp: Tryptophan
CBA: Cytometric bead array
F/B: Firmicutes/Bacteroidetes
PCA: Principal component analysis
ENS: Enteric nervous system
VN: Vagus nerve
CRF: Corticotropin-releasing factor
BNDF: Brain-derived neurotrophic factor
NMDA: *N*-Methyl-d-aspartic acid receptor
IAA: 3-Indoleacetic acid
IPA: Indolepropionic acid
CNS: Central nervous system
SHR: Steroid hormone receptor
ESRRA: Estrogen-related receptor α
IFN-γ: Interferon-γ
